# Targeted Glioblastoma Treatment via Synthesis and Functionalization of Gold Nanoparticles With De Novo–Engineered Transferrin-Like Peptides: Protocol for a Novel Method

**DOI:** 10.2196/49417

**Published:** 2023-08-11

**Authors:** Augusto Müller Fiedler, Michelle Medeiros, Haidi Dalinda Fiedler

**Affiliations:** 1 Department of Neurological Surgery University of Miami/Jackson Memorial Hospital Miami, FL United States; 2 National Institute of Science and Technology for Catalysis Department of Chemistry Federal University of Santa Catarina Florianópolis Brazil

**Keywords:** gold nanoparticles, glioblastoma, blood-brain barrier, transferrin-like peptides, drug delivery, brain tumor, neuro-oncology, chemotherapy, nanoparticle functionalization, pharmaceuticals

## Abstract

**Background:**

Glioblastoma multiforme (GBM) is an aggressive brain tumor with limited treatment options due to the blood-brain barrier’s (BBB’s) impedance and inherent resistance to chemotherapy. Gold nanoparticles (AuNPs) functionalized with transferrin-like peptides show promise in overcoming these challenges, enhancing drug delivery to the brain, and reducing chemotherapy resistance.

**Objective:**

The primary goal of this study is to establish a detailed protocol for synthesizing and stabilizing AuNPs, functionalizing them with de novo–engineered transferrin-like peptides, and conjugating them with the chemotherapeutic agent temozolomide. This strategy aims to improve drug delivery across the BBB and circumvent chemotherapy resistance. The secondary objective includes an assessment of the safety and potential for in vivo use of the synthesized nanoparticle complex.

**Methods:**

The proposal involves multiple steps with rigorous quality control of AuNP synthesis, stabilization with surfactants, and polyethylene glycol coating. The engineered transferrin-like peptides will be synthesized and attached to the AuNPs’ surface, followed by the attachment of temozolomide and O6-methylguanine-DNA methyltransferase inhibitors. The resulting complex will undergo in vitro testing to assess BBB penetration, efficacy against GBM cells, and potential toxicity.

**Results:**

Initial preliminary experiments and simulations suggest successful synthesis and stabilization of AuNPs and effective attachment of transferrin-like peptides. We propose peptide attachment verification using Fourier transform infrared spectroscopy and surface plasmon resonance. Additionally, we will conduct pH stability tests to ensure our functionalized AuNPs retain their properties in acidic brain tumor microenvironments.

**Conclusions:**

The proposed functionalization of AuNPs with de novo–engineered transferrin-like peptides represents a novel approach to GBM treatment. Our strategy opens new avenues for drug delivery across the BBB and chemotherapy resistance reduction. While we primarily focus on in vitro studies and computational modeling at this stage, successful completion will lead to further development, including in vivo studies and nanoparticle design optimization. This proposal anticipates inspiring future research and funding in neuro-oncology, presenting a potentially innovative and effective treatment option for GBM.

**International Registered Report Identifier (IRRID):**

RR1-10.2196/49417

## Introduction

Our previous work demonstrated that pH buffer solutions in biological systems, such as blood and intravascular fluid, are typically maintained at a pH of approximately 7.4 by a carbonic-bicarbonate ion buffering system (accounting for 64% of buffering capacity). However, human blood pH varies between 7.35 and 7.45, indicating that a shift of 0.10 is significant. The hemoglobin-oxyhemoglobin buffering system accounts for approximately 28% of total buffering capacity, making it a critical process in respiration. For medical purposes, it is possible to determine the percentages of dissolved oxygen, nitrogen, carbon dioxide, and carbon monoxide through gas-liquid chromatography or an elemental analyzer. This analyzer includes a flash combustion furnace, a Porapak chromatographic column, and a thermal conductivity detector.

Moreover, the blood pH buffer comprises systems of acidic and basic proteins (about 7% of the total buffering system) and monoacid phosphates or diacid phosphates (roughly 1%). Enzymes, the central chemical agents of life, catalyze essential reactions with high selectivity. However, they are usually sensitive to pH and can function optimally only within a rather narrow pH range. The acidity of aqueous solutions determines which metal ions are soluble, which ones may become hydrolyzed, or which ones may potentially precipitate. As such, the availability of metal ions in the blood is pH dependent [1-3].

Furthermore, the inherent acidity of the brain tumor microenvironment poses a significant challenge for nanoparticle (NP)-based therapies. Thus, pH stability testing is critical to ensure that our NPs maintain their structural integrity and functional capacity in the acidic conditions of the brain tumor microenvironment.

The global burden of glioblastoma multiforme (GBM), a highly aggressive brain tumor, continues to grow due to its resistance to conventional treatment options and difficulty in drug delivery across the blood-brain barrier (BBB) [4,5]. Significant research has been devoted to developing innovative strategies for GBM treatment. One such promising area is the application of gold nanoparticles (AuNPs) [6,7].

AuNPs are increasingly recognized for their unique properties, including their high surface area-to-volume ratio, modifiable surface, and biocompatibility, rendering them potential vehicles for drug delivery [6,8]. Moreover, the functionalized ability to cross biological barriers such as the BBB makes NPs particularly valuable for brain tumor treatment [9-11].

Simultaneously, transferrin, a naturally occurring iron transport protein, has been shown to assist in drug delivery across the BBB due to the high expression of the transferrin receptor on the BBB and cancer cells, including GBM cells [11]. Transferrin-like peptides, engineered from studies on natural transferrin, could potentially enhance the delivery of therapeutic agents to the brain when conjugated to AuNPs [12-14].

Temozolomide, a commonly used chemotherapeutic agent for GBM, struggles with drug resistance and inadequate delivery to the tumor site. Coating AuNPs with temozolomide, coupled with O6-methylguanine-DNA methyltransferase (MGMT) inhibitors to overcome resistance, might provide a more effective strategy for GBM treatment [4,9,15].

Given the potential synergy among AuNPs, transferrin-like peptides, temozolomide, and MGMT inhibitors, we propose the synthesis, stabilization, and functionalization of AuNPs with de novo–engineered transferrin-like peptides and the aforementioned chemotherapeutic agents [7,13,14]. This exploratory proposal aims to outline a novel protocol for a potentially more effective GBM treatment strategy, offering insights and considerations for future researchers in this field.

## Methods

### Synthesis and Characterization of AuNPs

AuNPs with a diameter between 160 Å and 200 Å and a neutral or slightly negative charge are synthesized and stabilized with surfactants. We characterize the NPs using electron microscopy and spectroscopy techniques to confirm their size, shape, and surface properties [16-18].

### Reagents

The following reagents are used: gold (III) chloride hydrate (HAuCl_4_; Sigma-Aldrich, lot MKBN2548V; 99.999% purity), sodium citrate dihydrate (Na_3_C_6_H_5_O_7_.2H_2_O; Sigma-Aldrich, batch #SLBG6645V; >99.0% purity), Milli-Q Nanopure water (doubly deionized water with a conductance of 5.6×10^–8^ Ω^–1^·cm^–1^ and pH 6.0-7.0 from a type D-4744 Nanopure deionization system or Purelab UHQ system to prepare standard and reagent solutions), all other reagents were of the best available analytical grade, and all vessels in contact with samples or reagents were cleaned by soaking in 5.8 M nitric acid (overnight) and repeatedly rinsed with deionized water before use.

### Equipment and Materials

The equipment and materials included are a scale (model AY220; Shimadzu), reaction flasks with a 100-mL mouth, oil bath (silicone+glassware), magnetic bar, a laminar flow hood (TROX-100), Heating and stirring plate (model C-MAG HS4; IKA), 50-mL volumetric flasks, 10-mL volumetric flask, 5-mL weighing beakers, spatulas (nonmetallic), a 0.45-μm filter (Chromafil Xtra CA-45/25; Macherey-Nagel), nitrile gloves, 1-mL micropipette, safety glasses, Pasteur pipettes, plastic syringes, Nalgene 10-mL, and 50-mL Teflon tubes.

### General Conditions

#### Beware of Glassware

The glassware must be previously cleaned with a 40% nitric acid solution and Milli-Q Nanopure water (see below Nota bene, Lab-203 rules for washing glassware) and then dried in the TROX-100 hood. Before the beginning of the experiments, the glassware is placed under the germicidal lamp of the TROX-100 hood for 1 hour. Regarding storage, glassware (class A) must be protected from environmental dust in duly clean, closed, and identified containers.

#### Nota Bene Glassware cleaning

The method used to clean the glassware is known as drag washing and consists of first washing the glassware with a strong stream of water and letting the tap water overflow when filling the containers. This procedure is repeated at least 5 times. Then, the glassware is immersed in 40% nitric acid for 24 hours and, thereafter, rinsed again with distilled and deionized water until all the acid is removed (at least 4 times, allowing the water to overflow).

#### Synthesis and Preparation of Solutions

The experiments were carried out at the Catalysis and Interfaces Science Laboratory (LACFI-203) with a controlled temperature of 23±1 °C. The preparation of solutions and the synthesis of NPs should be carried out in the TROX-100 hood (flow 41 and 34.5 m/second). Synthesis should be carried out in a silicone bath. Before starting the experimental procedure, the hood should be sanitized with alcohol, along with all the material that will be placed inside the chapel, for example, supports, micropipettes, and reagent jars.

### Experimental Procedure

#### Heating System

Silicone oil should be previously heated on a heating plate (selector position at 300). In approximately 1 hour, an ideal oil temperature between 110 °C and 120 °C for the experiment is achieved.

#### Preparation of 10 mL of a 1% (by Weight) Sodium Citrate Solution

Sodium citrate (0.1 g) should be weighed in a 5-mL beaker, completely dissolved in 2-3 mL of water, and transferred to a 10-mL volumetric flask with its volume completely adjusted with distilled water. Thereafter, the 1% sodium citrate solution should be filtered by weight in the 0.45-μm filter (Chromafil Xtra CA-45/25) using a sterile plastic syringe. The solution should be conditioned at room temperature.

#### Preparation of 50 mL of a 0.01% Gold Solution by Weight

HAuCl_4_ (0.0050 g) should be weighed in a 5-mL beaker, dissolved completely in 2-3 mL of distilled water, and transferred to a 50-mL volumetric flask. The beaker should be washed with distilled water, and this washing water is transferred to the volumetric flask to remove all HAuCl_4_ from the walls of the beaker. The volume of the volumetric flask is then completely adjusted with deionized water. The control of Au concentrations follows the methodology described by Fiedler et al [19].

#### Synthesis of AuNPs

AuNPs with a diameter between 160 Å and 715 Å are prepared as described by Frens [20].

Fifty milliliters of 0.01% gold solution (by mass) should be transferred to the synthesis flask together with a magnetic bar and then placed in a previously heated oil bath. A watch glass should be placed on the mouth of the synthesis flask. In approximately 20 minutes, the gold solution boils, and then the sodium citrate solution should be added as described in Table 1, with stirring (with the stirrer in position 3). Stirring should be carried out for 30 minutes. Thereafter, the heating and stirring should be turned off, and the solution should be allowed to cool to room temperature and transferred to a Teflon tube.

**Table 1:** Experimental data for the preparation of gold nanoparticles (AuNPs).

Nanoparticle	Sodium citrate (mL)	Diameter (Å)
AuNP size A	1.00	160
AuNP size B	0.75	245
AuNP size C	0.50	410
AuNP size D	0.30	715

#### Packaging

The solution must be conditioned in a Teflon tube previously washed (as shown in the *Preparation of 10 mL of a 1% [by Weight] Sodium Citrate Solution* section), duly identified at the room temperature of the LACFI-203, and protected from light.

#### Important Notes (0.01% [By Weight] Gold Solution)

Because it is highly hygroscopic, care should be taken when weighing HAuCl_4_. The weighted masses are shown in Table 2. The HAuCl_4_ reagent bottle must be stored in a desiccator [21].

**Table 2:** Weighted masses for the preparation of solutions.

Nanoparticle	Gold (III) chloride hydrate (g)
AuNP^a^ size A	0.0055
AuNP size B	0.0053
AuNP size C	0.0053
AuNP size D	0.0050

^a^AuNP: gold nanoparticle.

#### Preparation of 10 mL of a 1% (by Weight) Sodium Citrate Solution

The weight of sodium citrate should be 0.1004 g.

#### Synthesis of AuNPs

To measure the volume of sodium citrate, the micropipette aligned with the laboratory’s quality control was used. An error rate of 4% was tolerated, and the volume was 502.21 μL.

#### Functionalization of AuNPs

We propose coating AuNPs with polyethylene glycol to enhance their stability and biocompatibility [15,22]. Temozolomide, a chemotherapeutic agent, would be either attached to the surface of the AuNPs or could be encapsulated within them [4,12]. Additionally, the NPs’ design has to offer co-delivery of MGMT inhibitors to overcome resistance in GBM cells [7].

More data are needed to reveal the overall integrity of AuNPs post synthesis and post modification to validate successful incorporation of peptides onto AuNPs. Thus, verifying peptide attachment and assessing pH stability are fundamental steps prior to initiating in vitro experimental procedures.

#### Peptide Attachment Verification

After the synthesis and functionalization of the AuNPs, it is crucial to confirm the successful attachment of peptides onto the NPs. We propose a combination of Fourier transform infrared spectroscopy (FTIR) and surface plasmon resonance (SPR) for this verification.

FTIR can confirm peptide attachment by identifying the characteristic absorption peaks of the peptide-NP bond. After the peptide functionalized AuNPs, samples must be freeze-dried and mixed with potassium bromide. FTIR spectra must be collected in the range of 4000 cm^–1^ to 400 cm^–1^. The appearance of characteristic absorption bands in the FTIR spectrum, not present in the AuNPs without peptides, would confirm the successful attachment of peptides onto the NPs.

For SPR, we plan to coat a gold chip with a layer of the NPs and flow the peptide solution across the chip. The SPR sensor will monitor the real-time interaction between the NPs and the peptides, as reflected by changes in the resonance angle. A shift in the resonance angle upon the introduction of the peptide solution would confirm the binding of peptides to the AuNPs.

Both these methods can provide complementary information—FTIR would confirm the formation of a covalent bond between the AuNPs and peptides, while SPR will provide real-time information about the binding process and the stability of the peptide-AuNP complex.

#### pH Stability Test

Given the acidic nature of the tumor microenvironment, it is crucial to ensure that our NPs are stable and retain their functionality under such conditions. We propose conducting pH stability tests to tackle this problem. After verifying peptide attachment, NPs are incubated in solutions of varying pH (ranging from pH 4 to 8 to simulate physiological and tumor microenvironment conditions). We then evaluate the size, shape, and surface charge of the NPs through dynamic light scattering and zeta potential measurements, and the functionality of attached peptides is assessed using FTIR or SPR. This provides a comprehensive view of NP stability and functional integrity in a range of pH conditions.

#### In Vitro Evaluation

The functionalized AuNPs can be tested in vitro using GBM cell lines to evaluate their cellular uptake, cytotoxicity, and ability to overcome drug resistance [6]. As our current proposal is at an early stage, it focuses on in vitro studies and computational modeling. Upon successful completion of these initial stages, we plan to include in vivo animal studies in future protocols.

#### Optimization of NP Design

Based on the preliminary results, constant optimization of NP design is needed to enhance their mobility within the brain’s extracellular space, their penetration from the cerebrospinal fluid into the brain parenchyma, and their targeting efficiency [8]. Future developments in NP design optimization can involve adjusting NP size and shape to improve mobility and penetration, modifying surface properties for enhanced drug delivery and stability, and potentially incorporating targeting ligands that can increase affinity for GBM cells or specific receptors on the BBB. Each step represents a critical aspect of the research process and will contribute to the development of a more effective treatment for GBM. It is important to note that this complex and challenging project requires a multidisciplinary team of researchers with expertise in NP synthesis, drug delivery, cell biology, neuroscience, and oncology.

### Ethical Considerations

Since our proposal is centered on laboratory experiments and computational models, no human subjects or data from human subjects are involved in this research protocol. Therefore, human subjects research ethics review, informed consent, privacy and confidentiality protection, and compensation type and amounts for human subjects research are not applicable to our research proposal. However, should the study progress to the stage of clinical trials, all appropriate ethical considerations will be rigorously applied, including but not limited to obtaining ethical approval from the relevant institutional review board, obtaining informed consent from all participants, ensuring privacy and confidentiality, and providing fair compensation.

## Results

This proposal is currently theoretical and does not have reportable results other than structured simulations as listed above, where we found that the protocoled AuNPs have a structure capable of attaching transferrin-like peptides and carrying chemotherapeutics, such as temozolomide and MGMT inhibitors to overcome resistance. The authors do not anticipate proactively obtaining funding in the future due to insufficient resources.

## Discussion

### Limitations

While AuNPs are generally considered biocompatible, there could still be potential toxicity or adverse reactions, especially at the high doses for effective treatment [6]. The long-term effects of AuNPs’ accumulation in the body are not fully understood. Moreover, GBM tumors exhibit considerable heterogeneity, wherein various regions within a single tumor may display distinct characteristics [4]. This heterogeneity presents a significant challenge, potentially impeding the effectiveness of a singular therapeutic approach to treat the entire tumor.

As an early-stage proposal, our current focus is to establish the feasibility of our approach through computational models and in vitro studies. Moreover, we plan to generate data on AuNPs’ stability and delivery accuracy in subsequent studies.

### Comparison With Prior Work

In evaluating our proposal relative to recent literature [8], in particular, the study using radiolabeled AuNPs studded with substance P peptides for GBM treatment, it is clear that our approach offers several distinct advantages and complements existing strategies.

First, our work expands on the drug delivery capabilities of AuNPs. While the previous study used AuNPs for delivering radiation therapy, our nanocarrier approach delivers temozolomide and MGMT inhibitors, which are 2 crucial agents in chemotherapy for GBM [7]. The quality of a nanocarrier broadens the therapeutic range of AuNPs and illustrates their potential to transport various types of drugs.

Second, our study also emphasizes the importance of targeted delivery. Our nanocomplex approach (ie, AuNPs with transferrin-like peptides attached), seeks to exploit the upregulated transferrin receptor on GBM cells, thereby improving the specificity of drug delivery and minimizing off-target effects [12].

Another significant advancement in our study is the AuNPs’ ability to cross the BBB [4]. This passage is a crucial immune checkpoint and a key factor in neuro-oncologic therapies, which was not addressed in the previous study [8]. A successful BBB traversal by the functionalized AuNPs would suggest a promising strategy for delivering therapeutic agents to the central nervous system [7].

Furthermore, our study’s quality control and safety evaluation offers a comprehensive view of the potential impacts of the proposed treatment [6]. By investigating the toxicity of our compound, we could provide valuable insights into its suitability for further development and potential clinical application.

### Conclusions

The functionalization of AuNPs with de novo–engineered transferrin-like peptides presents a novel therapeutic approach for GBM. Our work proposes a detailed protocol explaining the synthesis, functionalization, and stability evaluation of AuNPs. Given the acidic nature of the brain tumor microenvironment, maintaining the structural integrity and functional capacity of AuNPs under these conditions is critical; hence, pH stability tests must be conducted accordingly. We demonstrate a potential modification to the surface of AuNPs to overcome current obstacles in GBM treatment. By introducing ligands or peptides—such as the proposed transferrin-like peptides—that target specific transporters or receptors on the BBB and show high affinity for GBM cells, we anticipate an enhanced ability to cross this barrier. Furthermore, we aim to enable accurate payload delivery by leveraging AuNPs as nanocarriers for temozolomide and MGMT inhibitors directly to the tumor site, potentially reducing chemotherapy resistance and systemic side effects. We hope that our rigorous quality control protocol for the synthesis of AuNPs will permit and inspire further research and potential funding in this field, offering an innovative and effective treatment option for GBM.

## Abbreviations

AuNP: gold nanoparticle
BBB: blood-brain barrier
FTIR: Fourier transform infrared spectroscopy
GBM: glioblastoma multiforme
LACFI-203: Catalysis and Interfaces Science Laboratory
MGMT: O6-methylguanine-DNA methyltransferase
NP: nanoparticle
SPR: surface plasmon resonance

## Data Availability

All data generated during this study are included in this published article. Data sharing is not applicable to this article as no data sets were generated or analyzed during this study.
